# Why do ineffective treatments seem helpful? A brief review

**DOI:** 10.1186/1746-1340-17-10

**Published:** 2009-10-12

**Authors:** Steve E Hartman

**Affiliations:** 1 Department of Anatomy, College of Osteopathic Medicine, University of New England, Biddeford, Maine 04005, USA

## Abstract

After any therapy, when symptoms improve, healthcare providers (and patients) are tempted to award credit to treatment. Over time, a particular treatment can seem so undeniably helpful that scientific verification of efficacy is judged an inconvenient waste of time and resources. Unfortunately, practitioners' accumulated, day-to-day, informal impressions of diagnostic reliability and clinical efficacy are of limited value. To help clarify why even treatments entirely lacking in direct effect can seem helpful, I will explain why real signs and symptoms often improve, independent of treatment. Then, I will detail quirks of human perception, interpretation, and memory that often make symptoms seem improved, when they are not. I conclude that healthcare will grow to full potential only when judgments of clinical efficacy routinely are based in properly scientific, placebo-controlled, outcome analysis.

## Why do ineffective treatments seem helpful?: A brief review

*"Much the greater part of medicine's useful and practical knowledge... derives not from physicians' observations of patients at the bedside but from the laboratories of the natural sciences, physics, and engineering"*[1].

An average day at the office: A patient presents with symptoms of a common, nonchronic malady. In your practice, you have observed this problem respond to a particular treatment protocol, and you manage the case accordingly. When your patient returns for follow up, symptoms are improved. Again, it seems your treatment has been effective.

In this contrived account, awarding credit to treatment seems reasonable, but is it? All we have are your personal, clinical impressions of cause and effect. For any particular case, your bedside experience and knowledge of an individual patient may inform diagnostic and treatment decisions, but are they likely to be enough?

Often, practitioners are tempted to base clinical convictions in personal experience. Controlled (scientific) verification of apparent efficacy can seem a bothersome hurdle. In fact, science sometimes seems to offer only a period at the end of a confident therapeutic sentence, already written. Unfortunately for those judging efficacy, symptoms can improve for many reasons unrelated to treatment. Even less-well understood by patients or practitioners, there are many reasons that symptoms may *seem *improved, when they are not. This assortment of causal possibilities renders casual, uncontrolled appraisals of clinical efficacy unreliable. In material that follows, I will show why:

1) clinical merits of one or more of your favored therapies might be open to question;

2) outcome studies must be designed and interpreted with caution;

3) randomized, placebo-controlled trials are the foundation of modern healthcare [2];

4) many medical journals (including this one) publish few case reports;

5) demonstrably valueless "alternative" and "complementary" approaches to healthcare are so popular; and

6) many alternative regimes (e.g., acupuncture, reflexology, and cranial osteopathy) seem effective against a plethora of health problems (from constipation and autism to Down Syndrome [3]) with numerous different biological foundations [3-5].

As we will see, discomfort engendered by opinions at odds with one's own can derail one's best intentions of reaching the truth. Although nothing in this review is, to my knowledge, new or controversial, some ideas expressed may conflict with views you already hold. If you give this review a thorough reading, and judge it incomplete (or even mistaken, in some regards), please consider publishing a counterview.

## History of Healthcare

*"The history of medicine has never been a particularly attractive subject... virtually anything that could be thought up for the treatment of disease was tried and, once tried, lasted decades or even centuries before being given up. It was, in retrospect, the most frivolous and irresponsible kind of human experimentation, based on nothing but trial and error and usually resulting in precisely that sequence." *[[6](p159)]

For as long as humans have struggled to heal other humans, confident practitioners (and trusting patients) have relied upon untold numbers of potions and procedures, shown (eventually) to be without value [7,8].

For millennia, to combat evil spirits or imbalance in their patients' four humors, caring, confident practitioner's drained copious quantities of blood [9]. This rarely led to desirable outcomes [8], and sometimes resulted in death [10].

For millennia, to repair imbalance or interruption of their patients' vital air or energy (chi or qi; pronounced "chee"), concerned healers have administered "traditional Chinese medicine." Herbal treatments, Quigong, acupuncture, moxibustion, pulse diagnoses, and an array of other folk medical therapies: all offered with conviction--and almost all have proven to be biologically vacuous [11,12].

For some maladies, healers have exploited environmental features superficially similar to symptoms. For example, yellow skin and eyes of jaundice may respond to a sip of water mixed with hair from a red bull [[13,14](Ch.III, Sec.2, p6)]. A tumor may "dry up" and disappear if a patient's neck is draped with part of a root from a specific plant, while the remainder is dried in smoke from an adjacent fire [[13,14](Ch.III, Sec.2, p7)]. Such practices draw their power from the precept "like cures like." This "doctrine of similarity" has been with us throughout recorded history. It provides much of the cognitive scaffold for magical thinking, in general [[13-16](p16)], and healthcare has suffered its share of embarrassing encounters.

Medical historians have recognized this lengthy, medical childhood of guesswork and therapeutic failure for what it was: a well-intentioned but desperately ill-informed struggle of our prescientific past [[6](p158-175), [7](p1-27),[8]]. For thousands of years, practitioners administered therapies, monitored symptoms, and then proclaimed their efforts beneficial. Patients considered their post-treatment perceptions, and agreed. Now we know, both practitioners and patients often were wrong. Whatever treatment was engaged, direct, positive effects on patient health probably were rare [[7](p1-27),[8]].

This lengthy record of misplaced medical confidence has done little to halt similar, 21^*st *^century errors in judgment. That is, universal acceptance of the evidence-based healthcare paradigm has faltered. Patients--and many practitioners--remain shackled by the same confounding factors that randomized trials, meta-analyses, and the Cochrane Collaboration [17] were designed to circumvent.

Use of some irrational practices has diminished, and rest harmlessly in the dust-bin of medical history (e.g., the cure-all of bloodletting). However, well-meaning practitioners still engage scientifically undetectable body energies of traditional Chinese medicine [11,12] and therapeutic touch [18]. Millions still cling to the long-discredited doctrine of similarities: are you familiar with homeopathy?

Why does faith in personal clinical experience persist, given its clear and protracted reputation for unreliability [7](p1-27) [8,19]? I consider three related components to this deductive malfunction:

**I**. due to natural history of disease, regression to the mean, and the placebo effect, real signs and symptoms often improve--with or without treatment;

**II**. patients and practitioners often convince themselves that treatment was effective--when it was not (due to confirmation bias and other human cognitive imperfections); and

**III**. personal evaluation of efficacy is quick and convincing, but properly controlled, scientific determinations can be slow, complex, and costly.

## I. Signs and symptoms improve, though treatment is without effect

Between initiation of therapy and any follow-up assessment, real, measurable improvement of symptoms often occurs--even if treatment is completely ineffective. How does this happen? Determining that partial credit should go to one's treatment requires prior elimination of other possibilities.

### Natural history of disease

Mammals have evolved immune systems, and other effective mechanisms for self-repair. Consequently, biologically real symptoms of disorder commonly diminish--no matter how (or whether) they are treated. Over time, a disease that naturally comes and goes may even provide patients and practitioners with *numerous *opportunities to assume--erroneously--that a treatment has been effective [20].

### Regression to the mean

Despite its statistically high-sounding label, regression to the mean is easily understood. Many clinical signs and symptoms, when measured through time, will be dispersed around a mean. Typically, patients seek care when symptomatic burdens of their dysfunction are greater than average. With or without treatment, subsequent measures of such properties likely will be closer to average (show improvement), for purely statistical reasons [21]. In fact, some researchers [22] believe that regression to the mean is responsible for most apparent improvements mistakenly assigned to the placebo effect. In other words, treatments often are not as powerful as we are inclined to believe, and the placebo effect isn't either.

### Placebo effect

Patients' interpretations of clinical encounters can inspire genuine health improvements [23,24](p127-165), [25-28](p25-43), [29-31]]. A practitioner's white coat, confident bearing, and gentle touch; a nurse's smile; a diploma covered wall; all can influence health-related physiology. These aspects of clinical outcomes, produced by the psychosocial context of any treatment (real or "placebo"), are called "placebo effects." Placebo-based improvements are real and important, but often are considered (instead) to be direct, clinical effects of treatment; this can only retard progress toward predictably, uniformly effective healthcare.

### Influences on health coincident with (but independent of) particular treatment

Initiative and frustration motivating patients to visit a particular healthcare provider may inspire other healthful actions, as well (e.g., behaviors leading to better diet, more sleep, more exercise, less stress, and therapies/medications supplied by other practitioners).

### *Post Hoc, Ergo Propter Hoc*

Not only can isolating real causes for clinical outcomes be a challenge, but also we seem *predisposed *to faulty reasoning. Why is credit so often (and erroneously) awarded to direct effect of a treatment, when it is the disease's natural history, regression to the mean, or a placebo effect that has brought real recovery?

In determining cause/effect relationships, a useful, cognitive rule-of-thumb takes the form: if event *B *follows event *A*, then *B *was caused by *A*. However, although cause does precede effect, hasty selection from among numerous preceding events is unwise. Nonetheless, the human proclivity to careless confidence in the temporal relationship between cause and effect is so strong that it long has been a subject of scholarly discourse. This cognitive flaw even has its own Latin label: *post hoc, ergo propter hoc *("after this, therefore because of this"). In a medical context, because real improvements often follow even an ineffective treatment, the treatment often is awarded credit when none is deserved. When practitioners and patients assume that improvements following treatment resulted directly *from *treatment, they often are being victimized by this cognitive fallacy.

## II. Symptoms do not improve, but seem to

Our abilities to perceive, interpret, and remember moment-to-moment experiences are limited [32-35]. In fact, we can be expected to mishandle some kinds of input. These limits to critical thinking are as likely to impair accurate comprehension of healthcare outcomes as they are any other life event. For example, the "*post hoc, ergo propter hoc*" fallacy often short-circuits critical thought, through the psychological phenomenon of "confirmation bias."

### Confirmation bias

*"People sometimes see ... patterns for which they are looking, regardless of whether the patterns are really there." *[[35](p181)]

*"A man hears what he wants to hear, and disregards the rest." *[36]

In 1951, Dartmouth College and Princeton University played a hard-fought game of (American) football, with penalties, animosity, and broken bones, in profusion. Subsequently, students from both campuses viewed a film of the entire game, and were asked to judge blame for the two teams' hostile behavior. Students from both schools assigned more guilt to members of *the other *team than suggested by recorded statistics [37]. Evidently, coincident with university affiliation, indoctrination, and allegiance, students expected members of their own team (who were, after all, the "good guys") to behave themselves. This motivated students to watch the other team more carefully, and they saw what they expected to see. Probably, these students' judgment was compromised by "confirmation bias" [35], the natural human inclination to gather, interpret, or remember information in ways that support preexisting desires or expectations [35,38].

#### Confirmation bias as a selective advantage

Many factors contribute to the mix of cognitive and behavioral phenomena labeled "confirmation bias" [35]. Though usually considered a cognitive flaw, confirmation bias may have been useful in environments of our evolutionary past. Seeking factual certainty at every life juncture may have been selectively unwise. Instead, certain time-saving, cognitive rules-of-thumb may have increased reproductive fitness [34,39]. Suppose one of your direct ancestors, while digging tubers, heard a stick break behind her. Your existence today might depend on her reaction: would you rather she exercised confirmation bias (honoring her triggered fear of predators with a jump to the nearest tree), or verified danger before burning precious calories?

#### Confirmation bias and cognitive dissonance

Cognitive dissonance [40] is "an unpleasant psychological state resulting from inconsistency between two or more elements in a cognitive system" [38]. Our proclivity to confirmation bias can be partly explained by our inclination to avoid cognitive dissonance.

By late 1954, Dorothy Martin was convinced a cataclysmic flood soon would inundate North America, and perhaps the entire world [41]. Fortunately for Dorothy and devoted Chicago-area converts to this parareligious conviction, technologically advanced beings of planet Clarion had (telepathically) promised safety. Prior to the December 21 reckoning, believers would be transported to another planet (or other safe place), in what we would call a "flying saucer."

December 21, 1954 came ... and went ... without aliens, space ships, or watery devastation. Did Dorothy and cohorts desist with proclamations of extraterrestrial beings and cosmic agendas? A few did. Most, however, rationalized the flood's absence, tweaked their odd convictions, and then proselytized with renewed vigor. That is, when facts diverged from fantasy, their faith held firm. Why?

Preparing for departure, many believers willingly had relinquished all belongings, and publicly had championed a bizarre view of reality. When predicted destruction and salvation failed to materialize, psychological options were few. Admitting they had abandoned possessions and reputations to a delusion would bring regret and embarrassment. Instead, avid believers apparently circumvented dissonant feelings of personal failure and shame by altering the fantasy to permit continued--even expanded--faith.

This is one of the earliest, now classical, published reports of how psychological discomforts of cognitive dissonance can lead individuals to rationalize and strengthen beliefs, rather than confront reality.

#### Confirmation bias and healthcare

Whatever its evolutionary and psychological roots, how does confirmation bias manifest in a patient's determination of whether a treatment has been helpful?

##### Desire

We *desire *treatment success because illness is unpleasant. If measurable signs/symptoms do not subside after treatment, patients can reduce at least their psychological burden by interpreting ambiguous symptoms positively, and by reporting (and believing) they feel better. We also may desire treatment success because failure could suggest poor (even foolish) investment of time and money. The temptation to deflect that dissonant reality (by confirming success, without evidence) may be substantial. This may be especially true if (in retrospect) the treatment now seems unusual--as many alternative remedies might.

##### Expectation

A patient's personal experience with, or knowledge of, a particular treatment may lead to an apparently informed *expectation *of therapeutic success. Trust in the healthcare system, a particular practitioner, or the body's natural healing abilities, also may warrant optimism. Perhaps to avoid cognitive dissonance that can come with being wrong, such expectations may lead patients to conclude that a particular therapeutic overture has had direct, positive results, when it has not.

Likewise, under some circumstances, we may be biased to confirm desires and expectations *of others*, because social norms seem to require it. Suppose a patient perceives her practitioner as a good healer (smart, well-trained, hard-working, confident, and compassionate). Under such circumstances, a social "norm of reciprocity" [32] embodied in "demand characteristics" [42] of a therapeutic encounter, may persuade her to rationalize treatment success. That is, she may report (and believe) that treatment was helpful--not because it was, but because her perception of her role as "patient" demands it of her.

Patients can avert psychological conflicts accompanying treatment failures (even surgical: [43]) by interpreting clinical findings and feelings optimistically, and presuming symptom relief. In addition, because treatment precedes imagined healing, the "after this, therefore because of this" fallacy helps justify assignment of (undeserved) credit to the treatment. Given all of these biasing predispositions, perhaps we should be surprised if patients did *not *fall prey to confirmation bias.

Practitioners also are victims of confirmation bias; cognitive dissonance; norms of reciprocity; the *post hoc, ergo propter hoc *fallacy; and other limits on the quality of human cognition [[19,35](p189,192-3),[44,45]]. For example: a practitioner's long, frequent, committed use of a particular technique or medication may put both professional and personal esteem "on the line." Thus, practitioners are at risk of dissonance every time earlier judgments of efficacy are reconsidered. Likewise, practitioners may be biased to confirm success--because their role as healer (rather than objective evidence) demands it. Unconscious temptations for practitioners to confirm desired/expected clinical outcomes may be as great as for patients.

#### Confirmation bias and self-fulfilling prophecy

After treatment, confident practitioners often query patients in ways that elicit answers validating practitioners' optimism, confirming hopes and expectations of both [46].

1) Practitioners sometimes achieve genuine treatment success. In addition, regression to the mean, placebo effects, confirmation bias, and the tendency for maladies to improve on their own lead to numerous *mistaken *impressions of efficacy. With so much perceived clinical triumph "under their belts," practitioners often begin therapeutic encounters *expecting *efficacy.

2) Expectations of success can lead practitioners (unknowingly) to ask questions, at follow-up, that guide patients to confirm treatment success, whatever their impressions before being questioned [46].

3) Hearing from patients what they expected, practitioners may become even more confident.

4) Perceiving practitioners' growing pleasure with apparent success, patients may become even more certain treatment was effective.

In this way, "self-fulfilling prophecies" [47,48] are interactively constructed by practitioners and patients. Not only can both reach erroneous conclusions on treatment success, but under many circumstances, they will create this shared delusion, together.

For many evolutionary, motivational, and cognitive reasons, humans are naturally disposed to confirmation bias. We are prone to observe, interpret, and remember events and information in ways that affirm both desires and expectations. In the context of healthcare, there are many reasons for both patients and practitioners to desire and expect treatment success, often prompting impressions that a treatment has been effective--when it has not.

### Peripheral factors that can relieve perceived symptoms

Numerous features of daily life, in general, can serve as psychological palliatives. In particular, a patient's interaction with one or more elements of the healthcare community may lead to reduced anxiety; feelings of increased control; or a positive, more realistic conception of the problem [32]. Any of these may inspire a patient to report symptom improvements--in the absence of real, physiological/anatomical treatment-related changes.

No matter how emphatically they praise you, patients' reports may tell you little about efficacy. Whatever your confidence that you have interpreted clinical outcomes with detachment, self-interest and limits of cognition may preclude you from doing so.

## Summary

Occasionally, purposeful clinical treatment leads directly to symptom improvement. More often, patients and practitioners award credit to a particular therapy when healing is unrelated (or even imaginary). Independent of any specific treatment, measurable signs and symptoms often improve, due to the self-correcting course of many diseases; regression to the mean; placebo effects; and other factors coincident with (but directly unrelated to) treatment. Even more worrisome, symptom improvement may be only imagined, consequent to various forms of confirmation bias, and peripheral factors leading to psychological palliation.

Any one of these confounding factors, by itself, renders uncontrolled judgments of direct clinical efficacy unreliable. Taken together, their influence is hard to overstate. When you treat a patient, apparent outcomes often will be influenced by real, physiological changes directly unrelated to treatment, and various forms of wishful thinking. Not only can these factors lead to erroneous perception of efficacy, but *they should be expected to*.

Only after formally controlled observations (limiting such biases) can practitioners be confident that clinical value of a treatment may have been accurately and reliably measured. A "no treatment" group helps control for a disease's natural history, regression to the mean, and some other factors leading to real change (in the absence of treatment). Remaining unrestrained will be real effects of placebos, and imaginary healing deriving from confirmation bias and other psychological influences. For these, inclusion of a sham treatment group offers the only hope of control. Only with good sham treatment, adequate sample size, random assignment of patients to study groups, and other precautions can direct effects of treatment (if any) be measured.

Independent of direct, effective, therapeutic support, patients often come to feel better. This is not trivial, but ethics of all healing professions demand that such effects not be falsely credited to specific treatments.

I hope I have engendered appreciation, even enthusiasm, for the importance of rigorously controlled clinical observation. Without science, healthcare still would involve little more than applying tourniquets, setting bones, and administering placebos. After many centuries as socially sanctioned, organized magical thinking, healthcare has been transformed by scientific inquiry into a powerful service profession. In fact, science has become integral to everything healthcare providers do. If you see patients, I hope you now will be suspicious about all assumptions of therapeutic success, including your own.

## Competing interests

The author declares that he has no competing interests.
